# The crystal structure of KScP_2_O_7_

**DOI:** 10.1107/S2056989020010427

**Published:** 2020-08-04

**Authors:** Günther J. Redhammer, Gerold Tippelt

**Affiliations:** a Chemistry and Physics of Materials, University of Salzburg, Jakob-Haringerstr. 2A, 5020 Salzburg, Austria

**Keywords:** KAlP_2_O_7_ structure type, pyrophosphate, scandium, isotypism, structure determination, crystal structure

## Abstract

Single crystals of KScP_2_O_7_, potassium scandium diphosphate, were grown in a borate flux. The title compound crystallizes isotypically with KAlP_2_O_7_ in space-group type *P*2_1_/*c*, *Z* = 4. The main building block is an {ScP_2_O_11_}^9–^ unit, forming layers parallel to (001). These layers are stacked along [001] *via* common corners of octa­hedral and tetra­hedral units to span up large hepta­gonal cavities that host the potassium cations with a coordination number of 10. The P—O—P bridging angle increases with increasing size of the octa­hedrally coordinated *M* ^III^ cation, as do the K—O distances within a series of K*M* ^III^P_2_O_7_ compounds (*M* ^III^ = Al to Y with ionic radii *r* = 0.538 to 0.90 Å).

## Abstract 2

The crystal structure of the pyrophosphate KScP_2_O_7_ crystallizes in the KAlP_2_O_7_ structure type and is compared with other structures of the K*M* ^III^P_2_O_7_ series (*M* = Al–Y).

## Chemical context   

Metal-phosphates with open framework structures raise large inter­est due to a rich crystal chemistry (Clearfield, 1988 ▸) and possible inter­esting applications, *e.g.* as non-linear optical materials, solid-state electrolytes, ionic conductors, battery materials or sensors (Hagerman & Poeppelmeier, 1995 ▸; Vītiņš *et al.*, 2000 ▸). In the context of solid-state electrolytes, we recently investigated the Na-super ionic conducting NaSICON-type compounds Na_3_Sc_2_(PO_4_)_3_ (NSP) and Ag_3_Sc_2_(PO_4_)_3_ (ASP) in terms of their structural phase-transition sequences and ionic conductivities (Rettenwander *et al.*, 2018 ▸; Ladenstein *et al.*, 2020 ▸; Redhammer *et al.*, 2020 ▸). To elucidate the role of the alkali metals on symmetry, we intended to synthesize the potassium analogue of NSP with flux-growth techniques. Using a method applied by Sljukic *et al.* (1967 ▸) for the synthesis of large crystals of NaSICON-type KZr_2_(PO_4_)_3_, however, did not yield the intended compound K_3_Sc_2_(PO_4_)_3_, but the title diphosphate KScP_2_O_7_ instead.

Vītiņš *et al.* (2000 ▸) reviewed that for such *A* ^I^ *M* ^III^P_2_O_7_ (*A* = Li, Na, K, Rb, Cs, Tl; *M* = Al, Ga, Fe, In, Sc, …) compounds six different structure types can be distinguished. They confirm that structure type I, involving compounds with large *A* site cations, is the largest group, showing *P*2_1_/*c* space-group symmetry. KAlP_2_O_7_ (Ng & Calvo, 1973 ▸) is regarded as the aristo-structure of group I with around 45 different compositions as compiled in the Inorganic Crystal Structure Database (ICSD; Zagorac *et al.*, 2019 ▸). For K-containing compounds, further materials are KCrP_2_O_7_ (Gentil *et al.*, 1997 ▸), KGaP_2_O_7_ (Genkin & Timofeeva, 1989 ▸), KFeP_2_O_7_ (Riou *et al.*, 1988 ▸; Genkin & Timofeeva, 1989 ▸), KVP_2_O_7_ (Benhamada *et al.*, 1991 ▸), KTiP_2_O_7_ (Zatovsky *et al.*, 2000 ▸), KMoP_2_O_7_ (Leclaire *et al.*, 1989 ▸; Chen *et al.*, 1989 ▸), KInP_2_O_7_ (Zhang *et al.*, 2004 ▸), KLuP_2_O_7_ (Yuan *et al.*, 2007 ▸), KYbP_2_O_7_ (Horchani-Naifer & Férid, 2007 ▸), KErP_2_O_7_ (Chaker *et al.*, 2016 ▸) and KYP_2_O_7_ (Yuan *et al.*, 2007 ▸). Synthesis and lattice parameters of Ce^III^-doped polycrystalline KScP_2_O_7_ as well as the luminescence properties were reported recently by Zhang *et al.* (2016 ▸); however, no atomic coordinates were given.

In this contribution, we present the determination of the crystal structure of KScP_2_O_7_, not reported so far, and compare it with the series of other K-containing diphosphates.

## Structural commentary   

**Figure 1:**
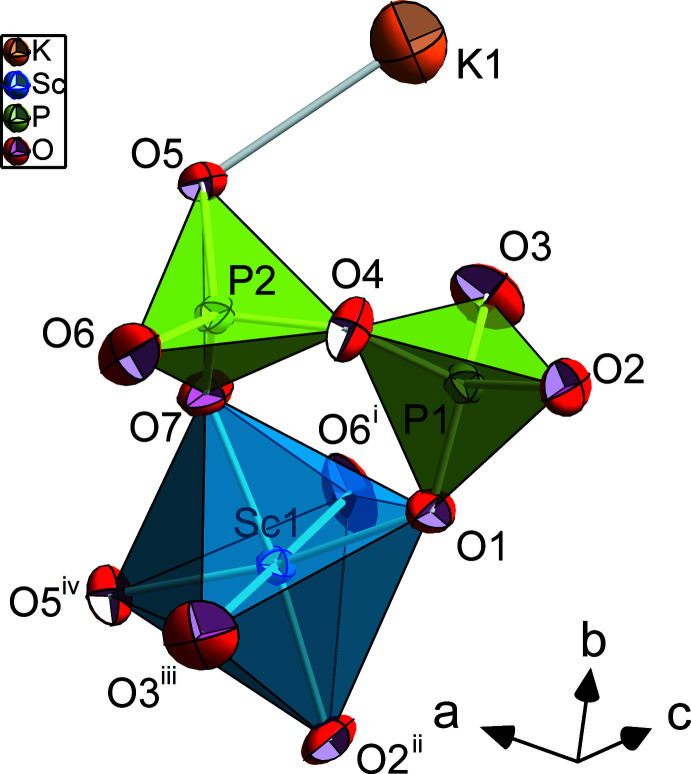
The principal building unit of KScP_2_O_7_ shown with displacement ellipsoids at the 95% probability level. [Symmetry codes: (i) *x*, 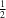 − *y*, 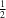 + *z*; (ii) 1 − *x*, −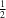 + *y*, 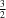 − *z*; (iii) *x*, 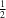 − *y*, −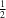 + *z*; (iv) 2 − *x*, −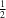 + *y*, 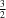 − *z*].

The title compound crystallizes in space group *P*2_1_/*c* and is isostructural with KAlP_2_O_7_ (Ng & Calvo, 1973 ▸). It contains one distinct K and Sc atom site, two distinct P atom and seven different oxygen-atom positions, all of them on general position 4 *e*. The basic building unit is a pyrophosphate group, which is formed by two distinct PO_4_ tetra­hedra (Fig. 1 ▸). They share the O4 oxygen atom, and the bridging P1—O4 and P2—O4 bond lengths are distinctly longer [1.6128 (6) and 1.6076 (6) Å, respectively] than the three shorter terminal P—O bonds [ranging between 1.4944 (7) and 1.5207 (6) Å]. These latter distances are those to the oxygen atoms which are shared with the ScO_6_ octa­hedra. The tetra­hedral O—P—O angles involving the bridging oxygen atom O4 are generally smaller, those involving the terminal oxygen atoms distinctly larger than the ideal O—*T*—O angle of 109.5°. This – together with the difference in bond lengths between bridging and non-bridging *T*—O bonds – induces polyhedral distortion (especially for the tetra­hedral angle variance, TAV), which is distinctly larger for the P1 tetra­hedron. Likewise, the average bond length is slightly larger for the P1O_4_ tetra­hedron than for the P2O_4_ tetra­hedron (Table 1 ▸). When comparing average bond lengths and polyhedral distortion parameters of the series of K*M* ^III^P_2_O_7_ structures (*M* = Al to Y), no clear variations with the ionic radius of the *M* cations can be found from the available data for tetra­hedral structure units and distortion parameters, and they remain almost constant. The parameters for KScP_2_O_7_ fit well into the data of the other K*M* ^III^P_2_O_7_ structures. The tetra­hedral bridging angle P1—O4—P2 amounts to 125.80 (5)° and is distinctly larger than that of KAlP_2_O_7_ [123.2 (11)°]. On the other hand, here a clear trend of increasing bridging angle with increasing size of the *M* cation is evident, *i.e.* the pyrophosphate group is stretched to account for the increase in size of the *M* cations (Table 1 ▸).

**Figure 2:**
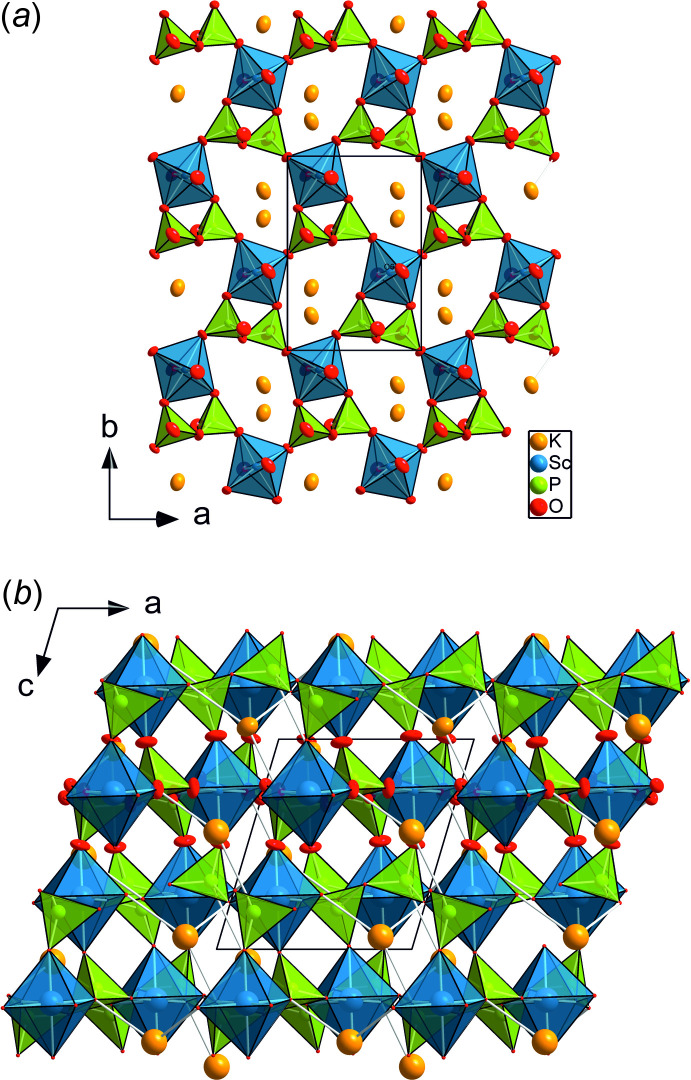
(*a*) Layer of laterally inter­connected {ScP_2_O_11_}^9−^ units, forming a layer parallel to (001), the K^I^ cations are hosted in the channels extending along [001]; (*b*) the three-dimensional framework structure of KScP_2_O_7_, viewed along [010].

The terminal oxygen atoms of the pyrophosphate group share their corners with five neighbouring ScO_6_ octa­hedra. Following Leclaire *et al.* (1989 ▸), two phosphate tetra­hedra and one octa­hedron form the basic {ScP_2_O_11_}^9–^ units (*cf*. Fig. 1 ▸), which are connected with units of the same kind *via* corner-sharing to make up a sheet parallel to (001), as depicted in Fig. 2 ▸. These layers are stacked along [001] in such a way that a ScO_6_ octa­hedron of one layer shares its O3^iii^ and O6^i^ corners (symmetry codes refer to Fig. 1 ▸) with one PO_4_ tetra­hedron each of the layer below and above.

**Figure 3:**
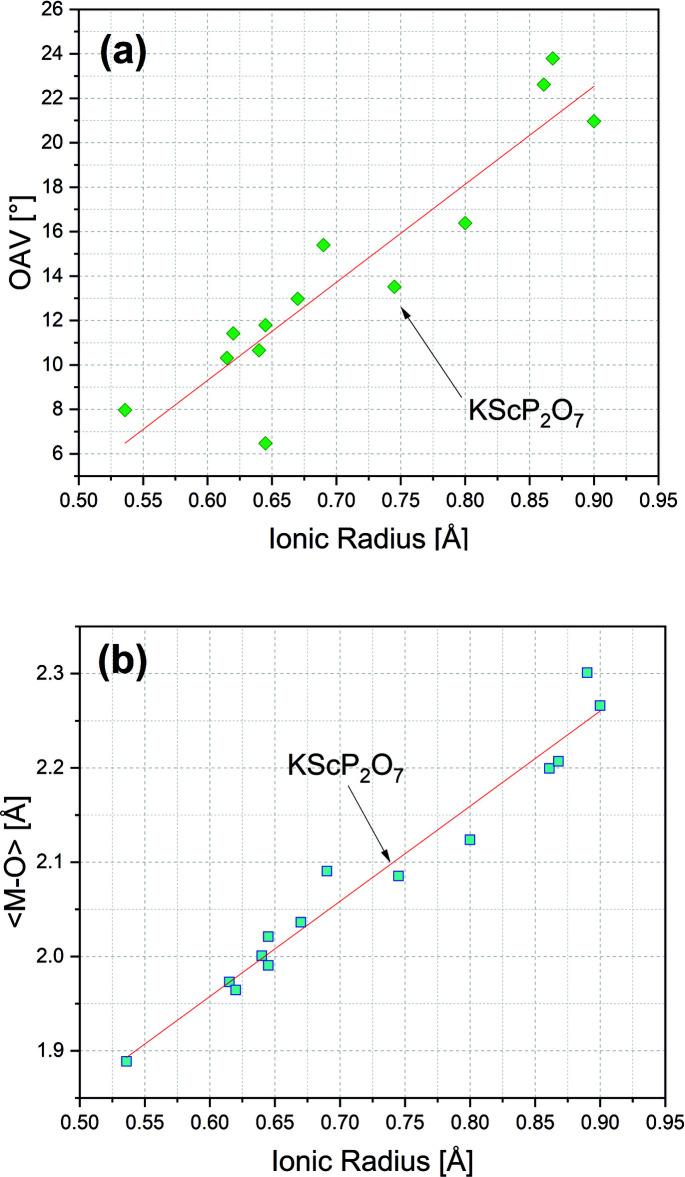
Variation of the octa­hedral angle variance (OAV) (*a*) and the average *M* ^III^—O bond lengths (*b*) as a function of the ionic radius of the *M* site cation across the series of K*M* ^III^P_2_O_7_ compounds (*M* = Al to Y).

Generally, all corners of the octa­hedron are shared with neighbouring PO_4_ tetra­hedra, whereby all oxygen atoms except O4 directly connect the octa­hedron with a pyrophos­phate P_2_O_7_ group, and the O2^ii^ and O5^iv^ oxygen atoms join the octa­hedron with two PO_4_ tetra­hedra within the above-mentioned layer parallel to (001). Additionally, the O1, O5^iv^ and O7 oxygen atoms (Fig. 1 ▸) are also bonded to one K^I^ cation each. The average Sc—O bond length is 2.085 Å while individual bond lengths range between 2.0736 (7) and 2.1122 (6) Å with one shorter bond (Sc—O6^i^) of 2.0346 (7) Å. A similar behaviour with one significantly shorter *M*—O bond is also observed in other K*M* ^III^P_2_O_7_ compounds and seems to be a more general feature. The Sc—O6^i^ and the Sc—O3^iii^ bonds, which point towards [001] and connect different (001) layers, both are the shortest within the ScO_6_ octa­hedron. Assuming that these two bonds are those to the axial oxygen atoms of the octa­hedron, the coordination polyhedron appears to be slightly compressed. Also, Ng & Calvo (1973 ▸) noted for KAlP_2_O_7_ that the axial bonds are considerably shorter that the equatorial ones within the (001) layer and – more generally speaking – this is also found in other K*M* ^III^P_2_O_7_ compounds. The ScO_6_ octa­hedron in the title compound is only slightly distorted in terms of bond lengths and bond-angle variance (Table 1 ▸). It is worth noting that KAlP_2_O_7_ shows the most regular octa­hedral coordination of all K*M* ^III^P_2_O_7_ structures compared here, and the distortion increases with increasing size of the octa­hedral cation as depicted in Fig. 3 ▸ *a*. The average <*M*—O> bond lengths also scale well with the ionic radius of the *M* site cation and are positively correlated (Fig. 3 ▸ *b*).

Large hepta­gonal cavities are formed in the skeleton of octa­hedral and tetra­hedral units that are made up from four tetra­hedrally and two octa­hedrally coordinated sites within the (001) layer. The stacking of the layers leads to channels running parallel to [001] where the potassium cations are hosted. They are tenfold coordinated with K—O bond lengths ranging between 2.7837 (7) Å and 3.3265 (9) Å, the average K—O bond length being 3.072 Å. As for <*M*—O>, the average K—O bond length also increases with increasing size of the *M* site cation, *i.e*. the channel size increases also.

**Figure 4:**
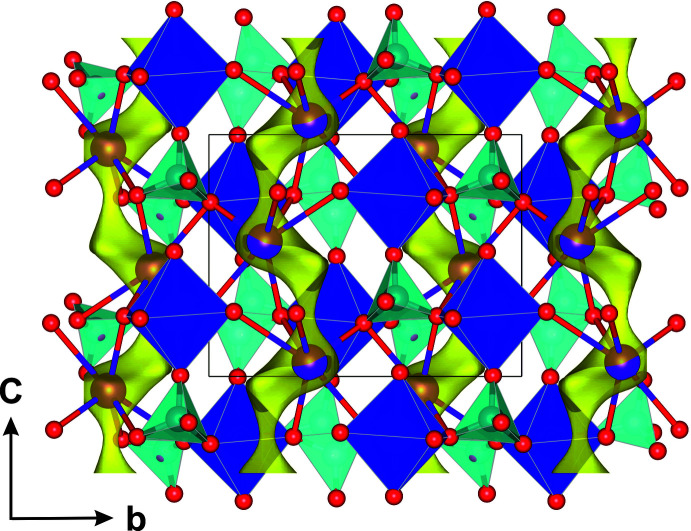
Bond-valence energy landscape map at levels of 1.5 eV above the minimum (yellow) viewed along [100]; the crystal structure of KScP_2_O_7_ is overlayed, the K sites lie within channels.

Using bond-valence energy landscape map (BVEL) calculations, an estimation of possible diffusion pathways of alkali ions in a compound can be facilitated. Using the program *SoftBV* (Chen & Adams, 2017 ▸; Chen *et al.*, 2019 ▸) such calculations were performed on KScP_2_O_7_ and reveal two energy minima. The lowest lying minimum is indeed occupied by the K^I^ cation, a second one is present at *x*, *y*, *z* = 0.271, 0.317, 0.438 (inter­stitial *i*1) and is unoccupied. A one-dimensional diffusion pathway is evident (Fig. 4 ▸), involving the *i*1 position, and is oriented parallel to [001]. An estimated activation energy of ∼0.3 eV would be needed to move a potassium ion from the regular K site to the inter­stitial *i*1 site; to move it from *i*1 to the next K1 site needs ∼1.3 eV. Inter­estingly, the percolation energy in *e.g*. Fe^III^, Mo^III^ and In^III^ compounds of the K*M* ^III^P_2_O_7_ series is distinctly higher with around 1.8 eV as estimated from BVEL maps. Generally, a partial substitution of trivalent cations by divalent ones might be of inter­est to increase the content of alkaline ions (here K^I^), which most probably could be found on the inter­stitial *i*1 site.

## Synthesis and crystallization   

The title compound was grown during attempts to synthesize NaSICON-type K_3_Sc_2_(PO_4_)_3_ adopting a flux growth protocol set up by Sljukic *et al.* (1967 ▸). Sc_2_O_3_ and KH_2_PO_4_ were mixed in stoichiometric qu­anti­ties (molar ratio 2:3) and B_2_O_3_ was added as a flux with a sixfold qu­antity of that of Sc_2_O_3_. The complete mixture was transferred to a platinum crucible, covered with a lid, and heated in a chamber furnace to 1473 K, held at this temperature for 24 h and then slowly cooled down to 1073 K at a rate of 3 K h^−1^. Between 1073 K and room temperature the cooling rate was 50 K h^−1^. The synthesis batch was immersed in hot water to dissolve the B_2_O_3_ and remaining K-phosphates. The residual contained single-phase KScP_2_O_7_ as checked by powder X-ray diffraction and showed single crystals of irregular to needle-like form with well-developed faces. The crystals are colorless and highly transparent with sizes up to 140 µm in lengths and ∼80 µm in diameter.

## Refinement   

Crystal data, data collection and structure refinement details are summarized in Table 2 ▸.

## Supplementary Material

Crystal structure: contains datablock(s) global, I. DOI: 10.1107/S2056989020010427/wm5578sup1.cif

Structure factors: contains datablock(s) I. DOI: 10.1107/S2056989020010427/wm5578Isup2.hkl

CCDC reference: 2019936

Additional supporting information:  crystallographic information; 3D view; checkCIF report

## Figures and Tables

**Table 1:** Selected structural and distortional parameters (Å, °) of K*M* ^III^P_2_O_7_ compounds Vol. = polyhedral volume (Å^3^), DI = distortion index, ECoN = effective coordination number, OQE = octa­hedral quadratic elongation, OAV = octa­hedral angle variance (°^2^), TQE = tetra­hedral quadratic elongation, TAV = tetra­hedral angle variance (°^2^). All calculations were performed using *VESTA* (Momma & Izumi 2011 ▸; for the mathematical meaning see the *VESTA Handbook*); ionic radii were taken from Shannon (1976 ▸).

*M*	Al	Cr	Ga	Fe^*a*^	Fe^*b*^	V	Ti	Mo	Sc	In	Lu	Yb	Er	Y
ionic radius	0.536	0.615	0.62	0.645	0.645	0.64	0.67	0.69	0.745	0.8	0.861	0.868	0.89	0.9
<K—O>	2.949	2.975	2.982	3.017	2.993	2.998	3.029	3.029	3.072	3.056	3.118	3.130	3.119	3.150
Vol.	44.03	45.21	45.23	50.08	45.73	46.03	47.21	47.63	49.40	48.45	52.04	52.11	52.09	53.25
DI	0.0442	0.0519	0.0484	0.0484	0.0535	0.0560	0.0596	0.0636	0.0620	0.0641	0.0695	0.0735	0.0813	0.0774
ECoN	8.84	8.38	8.54	8.58	8.29	8.22	7.89	7.51	7.66	7.29	7.09	6.63	6.80	6.40
														
<*M*—O>	1.889	1.973	1.964	2.021	1.991	2.000	2.036	2.091	2.085	2.124	2.199	2.207	2.301	2.266
Vol.	8.95	10.20	10.06	10.97	10.46	10.63	11.19	12.10	12.02	12.68	14.05	14.19	15.86	15.38
DI	0.0102	0.0091	0.0076	0.0313	0.0106	0.0154	0.0220	0.0086	0.0100	0.0073	0.0139	0.0116	0.0266	0.0074
OQE	1.0025	1.0031	1.0033	1.0032	1.0036	1.0036	1.0048	1.0045	1.0040	1.0047	1.0068	1.0070	1.0170	1.0060
OAV	7.97	10.31	11.42	6.47	11.79	10.66	12.98	15.40	13.52	16.38	22.62	23.79	54.07	20.97
ECoN	5.96	5.97	5.98	5.73	5.96	5.92	5.85	5.97	5.96	5.99	5.95	5.95	5.75	5.98
														
<P1—O>	1.536	1.542	1.540	1.537	1.537	1.540	1.540	1.536	1.537	1.538	1.545	1.531	1.494	1.518
Vol.	1.85	1.87	1.86	1.85	1.85	1.86	1.87	1.85	1.86	1.85	1.87	1.83	1.68	1.78
DI	0.0240	0.0241	0.0240	0.0124	0.0237	0.0245	0.0234	0.0256	0.0245	0.0281	0.0329	0.0264	0.0584	0.0326
TQE	1.0049	1.0053	1.0049	1.0042	1.0048	1.0047	1.0037	1.0052	1.0043	1.0059	1.0080	1.0043	1.0179	1.0074
TAV	19.55	21.29	19.79	17.39	18.95	18.34	14.44	20.41	16.80	22.82	27.35	16.12	37.83	24.70
ECoN	3.89	3.89	3.88	3.97	3.89	3.89	3.90	3.88	3.89	3.85	3.77	3.87	3.14	3.77
														
<P2—O>	1.531	1.535	1.535	1.530	1.531	1.535	1.536	1.534	1.535	1.530	1.546	1.535	1.509	1.529
Vol.	1.83	1.85	1.85	1.84	1.84	1.85	1.85	1.85	1.85	1.83	1.88	1.85	1.72	1.83
DI	0.0241	0.0242	0.0260	0.0088	0.0241	0.0227	0.0247	0.0231	0.0236	0.0272	0.0470	0.0237	0.0400	0.0351
TQE	1.0034	1.0032	1.0035	1.0011	1.0030	1.0026	1.0027	1.0027	1.0027	1.0034	1.0058	1.0024	1.0206	1.0038
TAV	12.54	11.68	12.48	4.49	10.67	9.18	9.12	9.60	9.48	10.99	9.51	7.94	71.43	4.61
ECoN	3.87	3.87	3.86	3.98	3.88	3.89	3.88	3.88	3.89	3.80	3.61	3.89	3.57	3.71
														
P1—O4—P2	123.18 (11)	123.68 (10)	123.8 (2)	n.d.	124.32 (10)	124.24	125.0 (2)	124.97 (15)	125.80 (5)	125.6 (5)	123.8 (9)	127.5 (3)	123.72 (7)	127.4 (6)

Notes: (*a*) data from Riou *et al.* (1988 ▸); (*b*) data from Genkin & Timofeeva (1989 ▸).

**Table 2:** Experimental details

Crystal data	
Chemical formula	KScP_2_O_7_
*M*_r_	258
Crystal system, space group	Monoclinic, *P*2_1_/*c*
Temperature (K)	293
*a*, *b*, *c* (Å)	7.4634 (1), 10.3902 (1), 8.3747 (1)
β (°)	106.49
*V* (Å^3^)	622.72 (1)
*Z*	4
Radiation type	Mo *K*α
μ (mm^−1^)	2.35
Crystal size (mm)	0.16 × 0.09 × 0.08

Data collection	
Diffractometer	Bruker SMART APEX CCD
Absorption correction	Multi-scan (*SADABS*; Krause *et al.*, 2015 ▸)
*T*_min_, *T*_max_	0.38, 0.52
No. of measured, independent and observed [*I* > 2σ(*I*)] reflections	20981, 2985, 2850
*R*_int_	0.021
(sin θ/λ)_max_ (Å^−1^)	0.837

Refinement	
*R*[*F* ^2^ > 2σ(*F* ^2^)], *wR*(*F* ^2^), *S*	0.018, 0.049, 1.05
No. of reflections	2985
No. of parameters	101
Δρ_max_, Δρ_min_ (e Å^−3^)	0.79, −0.66

Computer programs: *APEX2* and *SAINT* (Bruker, 2012 ▸), *SIR2014* (Burla *et al.*, 2005 ▸), *SHELXL2014/7* (Sheldrick, 2015 ▸), *DIAMOND* (Brandenburg, 2006 ▸), *ORTEP* for Windows and *WinGX* publication routines (Farrugia, 2012 ▸).

## supplementary crystallographic information

### Crystal data

**Table:**

KScP_2_O_7_	*F*(000) = 504
*M**_r_* = 258	*D*_x_ = 2.752 Mg m^−^^3^
Monoclinic, *P*2_1_/*c*	Mo *K*α radiation, λ = 0.71073 Å
Hall symbol: -P 2ybc	Cell parameters from 20981 reflections
*a* = 7.4634 (1) Å	θ = 2.9–36.5°
*b* = 10.3902 (1) Å	µ = 2.35 mm^−^^1^
*c* = 8.3747 (1) Å	*T* = 293 K
β = 106.49°	Prismatic, colorless
*V* = 622.72 (1) Å^3^	0.16 × 0.09 × 0.08 mm
*Z* = 4	

### Data collection

**Table:**

Bruker SMART APEX CCD diffractometer	2850 reflections with *I* > 2σ(*I*)
Graphite monochromator	*R*_int_ = 0.021
rotation, ω–scans at 4 different φ positions	θ_max_ = 36.5°, θ_min_ = 2.9°
Absorption correction: multi-scan (*SADABS*; Krause *et al.*, 2015)	*h* = −12→12
*T*_min_ = 0.38, *T*_max_ = 0.52	*k* = −17→17
20981 measured reflections	*l* = −13→13
2985 independent reflections	

### Refinement

**Table:**

Refinement on *F*^2^	Primary atom site location: structure-invariant direct methods
Least-squares matrix: full	Secondary atom site location: structure-invariant direct methods
*R*[*F*^2^ > 2σ(*F*^2^)] = 0.018	*w* = 1/[σ^2^(*F*_o_^2^) + (0.0257*P*)^2^ + 0.274*P*] where *P* = (*F*_o_^2^ + 2*F*_c_^2^)/3
*wR*(*F*^2^) = 0.049	(Δ/σ)_max_ = 0.001
*S* = 1.05	Δρ_max_ = 0.79 e Å^−^^3^
2985 reflections	Δρ_min_ = −0.66 e Å^−^^3^
101 parameters	Extinction correction: SHELXL2014/7 (Sheldrick 2015), Fc^*^=kFc[1+0.001xFc^2^λ^3^/sin(2θ)]^-1/4^
0 restraints	Extinction coefficient: 0.0429 (15)
0 constraints	

### Special details

**Table:**

Geometry. All esds (except the esd in the dihedral angle between two l.s. planes) are estimated using the full covariance matrix. The cell esds are taken into account individually in the estimation of esds in distances, angles and torsion angles; correlations between esds in cell parameters are only used when they are defined by crystal symmetry. An approximate (isotropic) treatment of cell esds is used for estimating esds involving l.s. planes.

### Fractional atomic coordinates and isotropic or equivalent isotropic displacement parameters (Å^2^)

**Table:**

	*x*	*y*	*z*	*U*_iso_*/*U*_eq_	
K1	0.82123 (3)	0.67829 (3)	0.94124 (3)	0.02342 (6)	
Sc1	0.76534 (2)	0.09952 (2)	0.74255 (2)	0.00607 (4)	
P1	0.55749 (3)	0.36164 (2)	0.80923 (2)	0.00673 (5)	
P2	0.86703 (3)	0.40292 (2)	0.67637 (3)	0.00681 (5)	
O1	0.54575 (9)	0.22058 (6)	0.76034 (9)	0.01132 (11)	
O2	0.36433 (9)	0.42041 (6)	0.76741 (9)	0.01313 (11)	
O3	0.67337 (11)	0.38889 (7)	0.98492 (9)	0.01712 (13)	
O4	0.66159 (9)	0.43502 (6)	0.69104 (8)	0.01167 (11)	
O5	0.99440 (9)	0.50481 (6)	0.77947 (8)	0.01003 (10)	
O6	0.85616 (11)	0.40831 (7)	0.49554 (8)	0.01632 (13)	
O7	0.92157 (9)	0.27011 (6)	0.75056 (8)	0.01088 (10)	

### Atomic displacement parameters (Å^2^)

**Table:**

	*U*^11^	*U*^22^	*U*^33^	*U*^12^	*U*^13^	*U*^23^
K1	0.02013 (10)	0.02973 (12)	0.01843 (10)	0.00408 (8)	0.00226 (7)	−0.00089 (8)
Sc1	0.00647 (6)	0.00539 (6)	0.00639 (6)	0.00013 (4)	0.00190 (4)	−0.00015 (4)
P1	0.00662 (8)	0.00633 (8)	0.00731 (8)	0.00074 (6)	0.00208 (6)	−0.00046 (6)
P2	0.00755 (8)	0.00703 (8)	0.00616 (8)	−0.00186 (6)	0.00246 (6)	−0.00017 (5)
O1	0.0091 (2)	0.0070 (2)	0.0185 (3)	0.00026 (18)	0.0050 (2)	−0.00208 (19)
O2	0.0086 (2)	0.0100 (2)	0.0214 (3)	0.00284 (19)	0.0052 (2)	−0.0002 (2)
O3	0.0209 (3)	0.0207 (3)	0.0073 (3)	0.0010 (3)	−0.0001 (2)	−0.0025 (2)
O4	0.0086 (2)	0.0124 (2)	0.0150 (3)	0.00151 (19)	0.0050 (2)	0.0054 (2)
O5	0.0103 (2)	0.0097 (2)	0.0107 (2)	−0.00398 (18)	0.00393 (19)	−0.00307 (18)
O6	0.0199 (3)	0.0229 (3)	0.0069 (2)	−0.0082 (2)	0.0049 (2)	−0.0016 (2)
O7	0.0098 (2)	0.0069 (2)	0.0159 (3)	−0.00059 (18)	0.0035 (2)	0.00091 (19)

### Geometric parameters (Å, º)

**Table:**

K1—O5	2.7837 (7)	P1—O3	1.5068 (7)
K1—O7^i^	2.7966 (7)	P1—O2	1.5123 (7)
K1—O1^ii^	2.8141 (7)	P1—O1	1.5176 (6)
K1—O7^iii^	2.9876 (7)	P1—O4	1.6128 (6)
K1—O5^i^	3.0259 (7)	P1—K1^vii^	3.5569 (3)
K1—O2^ii^	3.1505 (7)	P2—O6	1.4944 (7)
K1—O3	3.2592 (8)	P2—O5	1.5178 (6)
K1—O4	3.2835 (7)	P2—O7	1.5207 (6)
K1—O2^iv^	3.2927 (7)	P2—O4	1.6076 (6)
K1—O6^iii^	3.3265 (9)	P2—K1^i^	3.4856 (3)
K1—P2^i^	3.4856 (3)	P2—K1^viii^	3.6237 (3)
K1—P1^ii^	3.5570 (3)	O1—K1^vii^	2.8141 (7)
Sc1—O6^v^	2.0346 (7)	O2—Sc1^ii^	2.0883 (6)
Sc1—O3^vi^	2.0736 (7)	O2—K1^vii^	3.1505 (7)
Sc1—O2^vii^	2.0884 (6)	O2—K1^iv^	3.2927 (7)
Sc1—O5^viii^	2.0989 (6)	O3—Sc1^v^	2.0736 (7)
Sc1—O1	2.1045 (6)	O5—Sc1^iii^	2.0990 (6)
Sc1—O7	2.1122 (6)	O5—K1^i^	3.0259 (7)
Sc1—K1^viii^	3.9059 (3)	O6—Sc1^vi^	2.0346 (7)
Sc1—K1^vi^	3.9333 (3)	O6—K1^viii^	3.3265 (9)
Sc1—K1^i^	4.1501 (3)	O7—K1^i^	2.7966 (7)
Sc1—K1^vii^	4.2870 (3)	O7—K1^viii^	2.9875 (7)
			
O5—K1—O7^i^	106.35 (2)	O2^vii^—Sc1—K1^vi^	56.82 (2)
O5—K1—O1^ii^	108.45 (2)	O5^viii^—Sc1—K1^vi^	49.513 (18)
O7^i^—K1—O1^ii^	145.07 (2)	O1—Sc1—K1^vi^	135.284 (19)
O5—K1—O7^iii^	59.145 (18)	O7—Sc1—K1^vi^	118.557 (19)
O7^i^—K1—O7^iii^	93.300 (18)	K1^viii^—Sc1—K1^vi^	70.210 (7)
O1^ii^—K1—O7^iii^	106.983 (19)	O6^v^—Sc1—K1^i^	52.43 (2)
O5—K1—O5^i^	78.29 (2)	O3^vi^—Sc1—K1^i^	126.74 (2)
O7^i^—K1—O5^i^	50.565 (17)	O2^vii^—Sc1—K1^i^	140.66 (2)
O1^ii^—K1—O5^i^	135.51 (2)	O5^viii^—Sc1—K1^i^	79.508 (19)
O7^iii^—K1—O5^i^	113.314 (18)	O1—Sc1—K1^i^	94.313 (18)
O5—K1—O2^ii^	116.03 (2)	O7—Sc1—K1^i^	37.741 (18)
O7^i^—K1—O2^ii^	115.93 (2)	K1^viii^—Sc1—K1^i^	66.889 (5)
O1^ii^—K1—O2^ii^	48.919 (17)	K1^vi^—Sc1—K1^i^	128.501 (4)
O7^iii^—K1—O2^ii^	72.254 (18)	O6^v^—Sc1—K1^vii^	112.70 (2)
O5^i^—K1—O2^ii^	164.277 (19)	O3^vi^—Sc1—K1^vii^	67.21 (2)
O5—K1—O3	71.10 (2)	O2^vii^—Sc1—K1^vii^	75.012 (19)
O7^i^—K1—O3	103.820 (19)	O5^viii^—Sc1—K1^vii^	150.317 (19)
O1^ii^—K1—O3	84.749 (19)	O1—Sc1—K1^vii^	34.402 (18)
O7^iii^—K1—O3	130.140 (19)	O7—Sc1—K1^vii^	110.455 (18)
O5^i^—K1—O3	55.064 (17)	K1^viii^—Sc1—K1^vii^	131.220 (7)
O2^ii^—K1—O3	133.582 (19)	K1^vi^—Sc1—K1^vii^	101.144 (6)
O5—K1—O4	47.582 (17)	K1^i^—Sc1—K1^vii^	128.415 (5)
O7^i^—K1—O4	140.014 (19)	O3—P1—O2	113.29 (4)
O1^ii^—K1—O4	67.811 (18)	O3—P1—O1	114.72 (4)
O7^iii^—K1—O4	94.293 (17)	O2—P1—O1	110.44 (4)
O5^i^—K1—O4	90.703 (17)	O3—P1—O4	105.51 (4)
O2^ii^—K1—O4	103.768 (18)	O2—P1—O4	105.11 (4)
O3—K1—O4	44.629 (17)	O1—P1—O4	106.97 (4)
O5—K1—O2^iv^	120.669 (19)	O3—P1—K1^vii^	144.71 (3)
O7^i^—K1—O2^iv^	72.463 (19)	O2—P1—K1^vii^	62.23 (3)
O1^ii^—K1—O2^iv^	87.172 (19)	O1—P1—K1^vii^	49.33 (3)
O7^iii^—K1—O2^iv^	165.347 (19)	O4—P1—K1^vii^	109.38 (3)
O5^i^—K1—O2^iv^	54.972 (17)	O3—P1—K1	56.74 (3)
O2^ii^—K1—O2^iv^	116.65 (2)	O2—P1—K1	95.62 (3)
O3—K1—O2^iv^	53.305 (18)	O1—P1—K1	153.20 (3)
O4—K1—O2^iv^	94.610 (18)	O4—P1—K1	58.24 (3)
O5—K1—O6^iii^	97.331 (19)	K1^vii^—P1—K1	152.503 (8)
O7^i^—K1—O6^iii^	55.891 (18)	O6—P2—O5	113.29 (4)
O1^ii^—K1—O6^iii^	121.365 (19)	O6—P2—O7	112.29 (4)
O7^iii^—K1—O6^iii^	46.342 (17)	O5—P2—O7	110.41 (4)
O5^i^—K1—O6^iii^	100.309 (18)	O6—P2—O4	106.93 (4)
O2^ii^—K1—O6^iii^	72.478 (18)	O5—P2—O4	105.59 (4)
O3—K1—O6^iii^	153.88 (2)	O7—P2—O4	107.91 (3)
O4—K1—O6^iii^	140.332 (18)	O6—P2—K1^i^	140.37 (3)
O2^iv^—K1—O6^iii^	122.924 (18)	O5—P2—K1^i^	59.96 (3)
O5—K1—P2^i^	90.537 (15)	O7—P2—K1^i^	51.21 (3)
O7^i^—K1—P2^i^	25.078 (13)	O4—P2—K1^i^	112.48 (3)
O1^ii^—K1—P2^i^	150.579 (16)	O6—P2—K1^viii^	66.61 (3)
O7^iii^—K1—P2^i^	102.011 (14)	O5—P2—K1^viii^	104.90 (3)
O5^i^—K1—P2^i^	25.736 (12)	O7—P2—K1^viii^	53.74 (3)
O2^ii^—K1—P2^i^	140.943 (16)	O4—P2—K1^viii^	148.68 (3)
O3—K1—P2^i^	80.358 (14)	K1^i^—P2—K1^viii^	77.363 (6)
O4—K1—P2^i^	115.250 (14)	O6—P2—K1	126.27 (3)
O2^iv^—K1—P2^i^	63.593 (13)	O5—P2—K1	42.96 (2)
O6^iii^—K1—P2^i^	76.323 (13)	O7—P2—K1	121.13 (3)
O5—K1—P1^ii^	117.439 (16)	O4—P2—K1	62.66 (3)
O7^i^—K1—P1^ii^	131.596 (16)	K1^i^—P2—K1	77.719 (8)
O1^ii^—K1—P1^ii^	24.144 (13)	K1^viii^—P2—K1	146.911 (7)
O7^iii^—K1—P1^ii^	92.076 (14)	P1—O1—Sc1	127.58 (4)
O5^i^—K1—P1^ii^	154.610 (15)	P1—O1—K1^vii^	106.53 (3)
O2^ii^—K1—P1^ii^	25.133 (12)	Sc1—O1—K1^vii^	120.60 (3)
O3—K1—P1^ii^	108.821 (15)	P1—O2—Sc1^ii^	140.31 (4)
O4—K1—P1^ii^	87.268 (13)	P1—O2—K1^vii^	92.64 (3)
O2^iv^—K1—P1^ii^	99.950 (13)	Sc1^ii^—O2—K1^vii^	124.31 (3)
O6^iii^—K1—P1^ii^	97.304 (14)	P1—O2—K1^iv^	106.05 (3)
P2^i^—K1—P1^ii^	151.985 (9)	Sc1^ii^—O2—K1^iv^	91.12 (2)
O6^v^—Sc1—O3^vi^	178.96 (3)	K1^vii^—O2—K1^iv^	87.202 (17)
O6^v^—Sc1—O2^vii^	91.12 (3)	P1—O3—Sc1^v^	162.86 (5)
O3^vi^—Sc1—O2^vii^	89.85 (3)	P1—O3—K1	100.52 (4)
O6^v^—Sc1—O5^viii^	91.86 (3)	Sc1^v^—O3—K1	92.33 (3)
O3^vi^—Sc1—O5^viii^	88.54 (3)	P2—O4—P1	125.80 (4)
O2^vii^—Sc1—O5^viii^	88.65 (3)	P2—O4—K1	91.56 (3)
O6^v^—Sc1—O1	89.20 (3)	P1—O4—K1	97.07 (3)
O3^vi^—Sc1—O1	90.26 (3)	P2—O5—Sc1^iii^	133.60 (4)
O2^vii^—Sc1—O1	100.03 (3)	P2—O5—K1	115.23 (3)
O5^viii^—Sc1—O1	171.24 (3)	Sc1^iii^—O5—K1	105.40 (2)
O6^v^—Sc1—O7	89.01 (3)	P2—O5—K1^i^	94.31 (3)
O3^vi^—Sc1—O7	90.06 (3)	Sc1^iii^—O5—K1^i^	98.65 (2)
O2^vii^—Sc1—O7	173.98 (3)	K1—O5—K1^i^	101.71 (2)
O5^viii^—Sc1—O7	85.32 (2)	P2—O6—Sc1^vi^	163.73 (5)
O1—Sc1—O7	86.00 (2)	P2—O6—K1^viii^	89.04 (3)
O6^v^—Sc1—K1^viii^	110.60 (2)	Sc1^vi^—O6—K1^viii^	98.58 (3)
O3^vi^—Sc1—K1^viii^	69.08 (2)	P2—O7—Sc1	131.85 (4)
O2^vii^—Sc1—K1^viii^	125.45 (2)	P2—O7—K1^i^	103.71 (3)
O5^viii^—Sc1—K1^viii^	43.401 (17)	Sc1—O7—K1^i^	114.72 (3)
O1—Sc1—K1^viii^	128.378 (19)	P2—O7—K1^viii^	102.03 (3)
O7—Sc1—K1^viii^	49.150 (18)	Sc1—O7—K1^viii^	98.52 (2)
O6^v^—Sc1—K1^vi^	125.03 (2)	K1^i^—O7—K1^viii^	100.37 (2)
O3^vi^—Sc1—K1^vi^	55.89 (2)		

Symmetry codes: (i) −*x*+2, −*y*+1, −*z*+2; (ii) −*x*+1, *y*+1/2, −*z*+3/2; (iii) −*x*+2, *y*+1/2, −*z*+3/2; (iv) −*x*+1, −*y*+1, −*z*+2; (v) *x*, −*y*+1/2, *z*+1/2; (vi) *x*, −*y*+1/2, *z*−1/2; (vii) −*x*+1, *y*−1/2, −*z*+3/2; (viii) −*x*+2, *y*−1/2, −*z*+3/2.
